# Virus goes viral: an educational kit for virology classes

**DOI:** 10.1186/s12985-020-1291-9

**Published:** 2020-01-31

**Authors:** Gabriel Augusto Pires de Souza, Victória Fulgêncio Queiroz, Maurício Teixeira Lima, Erik Vinicius de Sousa Reis, Luiz Felipe Leomil Coelho, Jônatas Santos Abrahão

**Affiliations:** 1 Laboratório de Vírus, Departamento de Microbiologia, Instituto de Ciências Biológicas, Universidade Federal de Minas Gerais, Av. Antônio Carlos, 6627, Belo Horizonte, 31270-901 Brazil; 2 Laboratório de Vacinas, Departamento de Microbiologia e Imunologia, Instituto de Ciências Biomédicas, Universidade Federal de Alfenas, Rua Gabriel Monteiro da Silva, 700, Alfenas, 37130-001 Minas Gerais Brazil

**Keywords:** Virology, Virology education, Microbiology education, biology education, Didactic material, Giant viruses, Mimivirus, Poxvirus, Flavivirus, Alphavirus

## Abstract

**Background:**

Viruses are the most numerous entities on Earth and have also been central to many episodes in the history of humankind. As the study of viruses progresses further and further, there are several limitations in transferring this knowledge to undergraduate and high school students. This deficiency is due to the difficulty in designing hands-on lessons that allow students to better absorb content, given limited financial resources and facilities, as well as the difficulty of exploiting viral particles, due to their small dimensions. The development of tools for teaching virology is important to encourage educators to expand on the covered topics and connect them to recent findings. Discoveries, such as giant DNA viruses, have provided an opportunity to explore aspects of viral particles in ways never seen before. Coupling these novel findings with techniques already explored by classical virology, including visualization of cytopathic effects on permissive cells, may represent a new way for teaching virology. This work aimed to develop a slide microscope kit that explores giant virus particles and some aspects of animal virus interaction with cell lines, with the goal of providing an innovative approach to virology teaching.

**Methods:**

Slides were produced by staining, with crystal violet, purified giant viruses and BSC-40 and Vero cells infected with viruses of the genera *Orthopoxvirus*, *Flavivirus*, and *Alphavirus*. Slides with amoebae infected with different species of giant viruses and stained with hemacolor reagents were also produced.

**Results:**

Staining of the giant viruses allowed better visualization of the viral particles, and this technique highlights the diversity in morphology and sizes among them. Hemacolor staining enabled visualization of viral factories in amoebae, and the staining of infected BSC-40 and Vero cell monolayers with crystal violet highlights plaque-forming units.

**Conclusions:**

This kit was used in practical virology classes for the Biological Sciences course (UFMG, Brazil), and it will soon be made available at a low-cost for elementary school teachers in institutions that have microscopes. We hope this tool will foster an inspiring learning environment.

## Background

Viruses are the most numerous entities on Earth, and they are found in the majority of ecosystems [1]. Over a century after their discovery, viruses are often recognized by the population as pathogens associated with diseases and epidemics. They generate fear and fascination once they directly influence human life [2]. The study of viruses is known as virology; this subject is often considered to be a part of microbiology. In the early years, strong background in this discipline was essential for studying medicine and biology. Virology pedagogy presents several limitations and challenges, including a high monetary cost for materials and biosafety laboratory requirements. Additionally, students work with biohazardous materials that can endanger themselves and their colleagues [3].

The small size of viruses is a major obstacle that limits the study of virology. Therefore, learning viral morphology is often limited to electron microscopy figures and schematic illustrations of viruses [2]. This obstacle, however, became obsolete after the discovery of the *Acanthamoeba polyphaga* mimivirus (APMV), the first described amoeba giant virus [4–6]. APMV is the prototype species of the genus *Mimivirus* (4); it belongs to the nucleocytoplasmic large DNA viruses (NCLDV) [7–9]. Following the discovery of APMV, other giant viruses were described and characterized, including cedratvirus (CVG), pandoravirus (PDV), kaumoebavirus (KAUV), Orpheovirusbrasiliensis (OBRV), faustovirus (FSTV), and tupanvirus (TPV) [10–17]. Additionally, with the discovery of giant viruses, one of the paradigms of virology was broken, namely that viruses are considered to be “filterable organisms”. This shift led researchers to think that a portion of the virosphere is trapped in filters and begin research on prospection and characterization of giant viruses [18].

Another key point in virology is the observation of cytopathic effects (CPEs) [19]. CPEs refer to structural changes in host cells that are caused by the viral invasion. Some viruses cause characteristic CPEs, and observation of these effects is an important tool for virologists concerned with isolating and identifying viruses [19, 20]. From an educational perspective, myriad CPEs are visible by students under the optical microscope, such as changes in cell morphology, inclusion bodies, and lysis plaques [2, 19, 20]. Many medically important animal viruses show these effects over the course of infection [20]. In this study, we present the CPEs of poxviruses (vaccinia virus [VACV] and cowpox virus [CPXV] and arboviruses (yellow fever virus [YFV], chikungunya virus [CHIKV], and mayaro virus [MAYV]) on materials that are safe for classroom application. Besides, we used a wide range of giant virus preparations where particles and other viral structures as well as CPEs can be visualized.

Practical activities in biology provide opportunities for students to perform science rather than only learn about it. This modality allows the educator to expand topics in the classroom and connect them to recent discoveries [21, 22]. Some studies highlight the importance of combining laboratory and theoretical courses to attain a deeper understanding and greater satisfaction [23, 24]. Our goal in the present work was to develop an innovative way to address the concrete aspects of viral particle and host cell effects. This method, from an educational perspective, combines hands-on experience with a classroom virology course to allow the instructor to expand the students (and her/his own) knowledge. Thus, the purpose of our “Virus Goes Viral” kit was to explore the revolutionary aspect of giant viruses and also the classically explored aspects of animal virus interactions with cell lines and integrate them into the study of virology.

## Methods

### Virus and cells

TPV, CVG, PDV, and Niemeyer virus (NYMV) were propagated individually using *Acanthamoeba castellanii* (American Type Culture Collection [ATCC] 30010) cultured at 32 °C in 175 cm^2^ cell culture flasks with 50 mL peptone-yeast extract with glucose (PYG) medium supplemented with 25 mg/ml amphotericin B (Fungizone; Cristalia, São Paulo, Brazil) and 500 U/ml penicillin (Schering-Plough, Brazil). The amoebae were infected with at a multiplicity of infection (MOI) of 0.01. Cells were incubated until the expected CPEs appeared in culture, and the media was collected by two centrifugation steps. *A. castellanii* cells and cellular debris were first removed by centrifugation (400 x g for 10 min), and the particles were purified by centrifugation (36,000 x g for 1 h) through a sucrose cushion (40–50%), suspended in PAS, and stored at − 80 °C. KAUV, OBRV, and FTSV were propagated individually in *Vermamoeba vermiformis* (ATCC50237) cultivated at 32 °C in PYG medium with a MOI of 0.01. After the appearance of CPEs, the cells and supernatants were collected, with sterile serological pipettes, stored in sterile conical tubes, and the viruses were purified through ultracentrifugation with a sucrose cushion.

African green monkey kidney BSC-40 cells (ATCC CRL-2761) and Vero cells (ATCC CCL-81) were maintained in an atmosphere with 5% CO_2_ at 37 °C in Eagle’s minimum essential medium (MEM; Gibco BRL, Invitrogen, Carlsbad, CA, United States), supplemented with 5% fetal bovine serum (FBS; Cultilab, Brazil), 2.5 μg/mL amphotericin B, 500 U/mL penicillin (Cristalia), and 50 μg/mL streptomycin (Schering-Plough, São Paulo, Brazil).

YFV (vaccine strain 17DD), MAYV (Strain BeAr20290), CHIKV (Genotype ECSA – Strain BHI3762/H804917, kindly provided by Dr. Maurício Lacerda Nogueira), VACV group I (Isolate Caragola eye I), VACV group II (Isolate Caragola eye II), and CPXV (strain Brighton Red) were individually multiplied in Vero cells in MEM that contained 1–2.5% FBS, 0.25 μg/mL amphotericin B, 100 U/mL penicillin 100 U/mL (Schering-Plough, Brazil), and 100 μg/mL streptomycin at 37 °C in an atmosphere with 5% CO_2_ in a large 175 cm^2^ bottle. Subsequently, for YFV, MAYV, and CHIKV, the cell infection supernatant was transferred to tubes and centrifuged at 3,000 x g for 5 min. The clarified supernatants from these centrifugations were stored at − 70 °C. VACV and CPXV were purified on a sucrose gradient as previously described [25] and stored at − 70 °C.

### Slide preparation for microscopic visualization

#### Viral particles

Aliquots of purified virus were diluted 1:10 (CVG, OBRV, PdV, and TPV) or 1:20 (NYMV), and 10 μL of the appropriate dilution was placed on the center of glass slide. The liquid was spread using circular movements to obtain an approximately 1-cm diameter drop. The slide was kept at room temperature until the liquid dried on the surface. Subsequently, the virus was fixed by adding 200 μL methanol over the center of the slide and stained with crystal violet for 15 min. After staining, the slides were washed in running water and dried at room temperature. Once dried, the stained region was covered by a 13 mm glass coverslip and affixed to the slide with Canada balsam (Synth, Brazil).

#### Viral factories

Approximately 1 million *A. castellanii* cells in PYG medium were cultured in cell culture flasks (25 cm^2^). After the amoebae adhered to the flask, NYMV was inoculated with a MOI of 1. After 12-h infection, cells were removed from the flask and centrifuged at 885 x g for 10 min and stained with hemacolor reagents (Renylab, Brazil). Once dried, the stained region was covered with a 13 mm glass coverslip using Canada balsam.

#### Plaque-forming units

Two cell lines were used for visualization of virus plaque-forming units. For MAYV, CHIKV, and YFV, Vero cells (2 × 10^5^ cells/well) were plated on sterile 13 mm coverslips, grown in MEM with 5% FBS for 24 h, and maintained at 37 °C in an atmosphere with 5% CO_2_. The next day, the cells were infected with viruses and observed until the characteristic CPE appeared (lysis plaques). Once CPEs were detected, the coverslips were fixed in formaldehyde (3.7% v/v) for 2 h and stained with crystal violet. After staining, the coverslips were washed, dried, and affixed to the glass slides using Canada balsam. For viruses from both VACV groups and CPXV, lysis plaque visualization was performed by infecting BSC-40 cells. The same procedure described above was followed to prepare this slide.

#### Inclusion bodies

BSC-40 cells (2 × 10^5^ cells/well) were plated on sterile 13 mm coverslips, grown in MEM with 5% FBS for 24 h, and maintained at 37 °C in an atmosphere that contained 5% CO_2_. The cells were infected with CPXV (MOI: 10), and once CPEs were detected, the coverslips were fixed in formaldehyde (3.7% v/v) for 2 h and stained with the solution rich in eosin from the hemacolor kit for 10 min. After staining, the coverslips were washed, dried, and affixed to glass slides using Canada balsam.

## Results

### Viral particle visualization slides

**Fig. 1:**
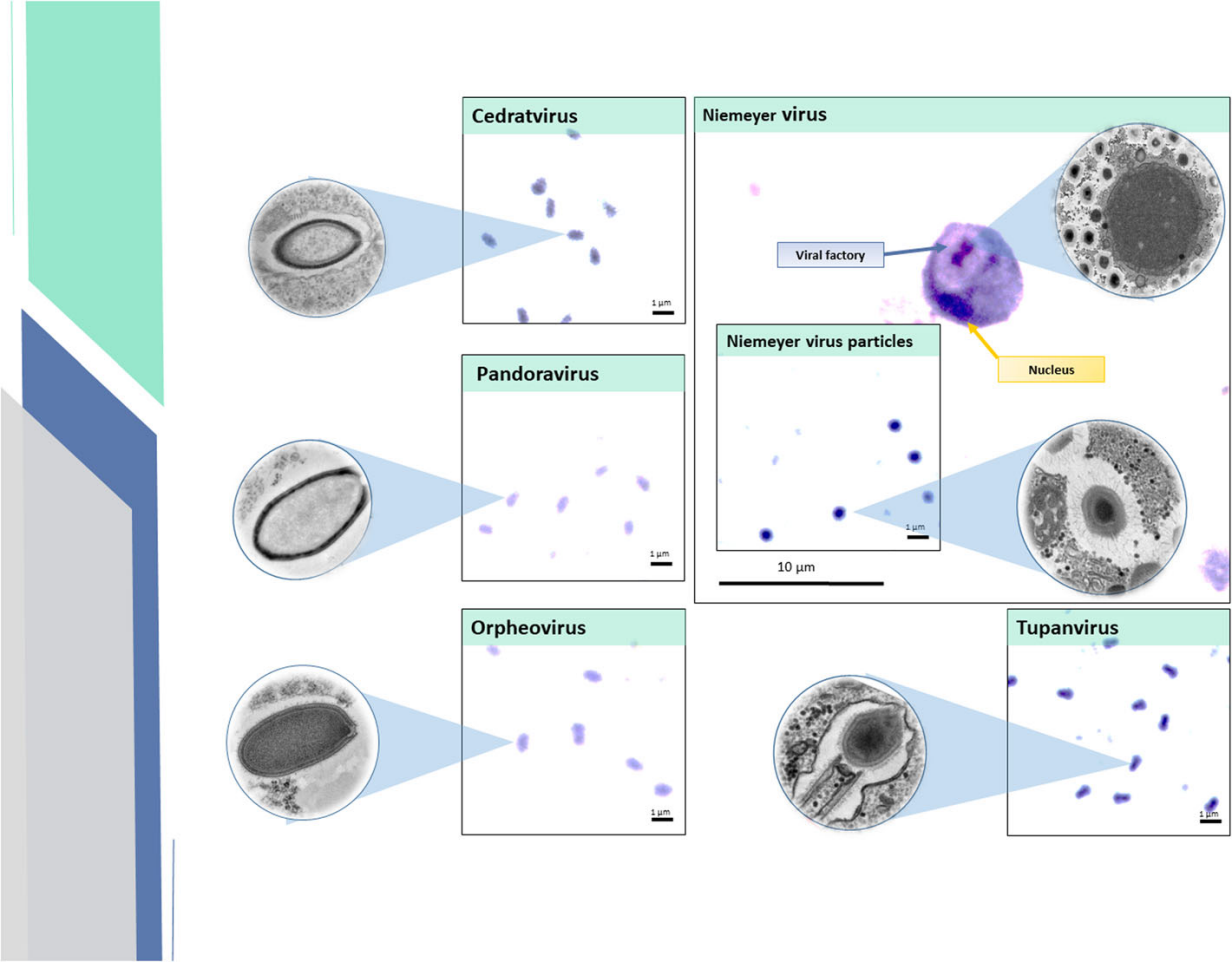
Presentation of different giant virus particles (cedratvirus, pandoravirus, orpheovirus, Niemeyer virus and tupanvirus) and viral factories of Niemeyer virus under light microscopy. Visualization and comparison of optical and electron micrographs for visualization of viral morphology and factories. Total magnification is 1000x

The slides produced with the purified viral particles stained with crystal violet allowed a simple evaluation of the distinct morphology and particle sizes for each isolate (Fig. 1). CVG, OBRV, and PDV particles were ovoid and measured ~ 1 μm in size and ~ 0.5 μm in diameter (Fig. 1). NYMV particles were ~ 0.6 μm and appeared spherical when viewed with optical microscopy. TPV particles were large and strongly stained with crystal violet. They appeared to have a spherical head and a cylindrical tail under optical microscopy (Fig. 1).

### Viral factory visualization slides

Viral factories were visualized after staining the cells with the hemacolor kit. The *Mimivirus* viral factory, such as for NYMV, is very identifiable, and was represented by a light halo with a strongly marked dark purple center located in the cell cytoplasm (Fig. 1, blue arrow). The nucleus was stained dark purple (Fig. 1, yellow arrow).

### CPE visualization slides

**Fig. 2:**
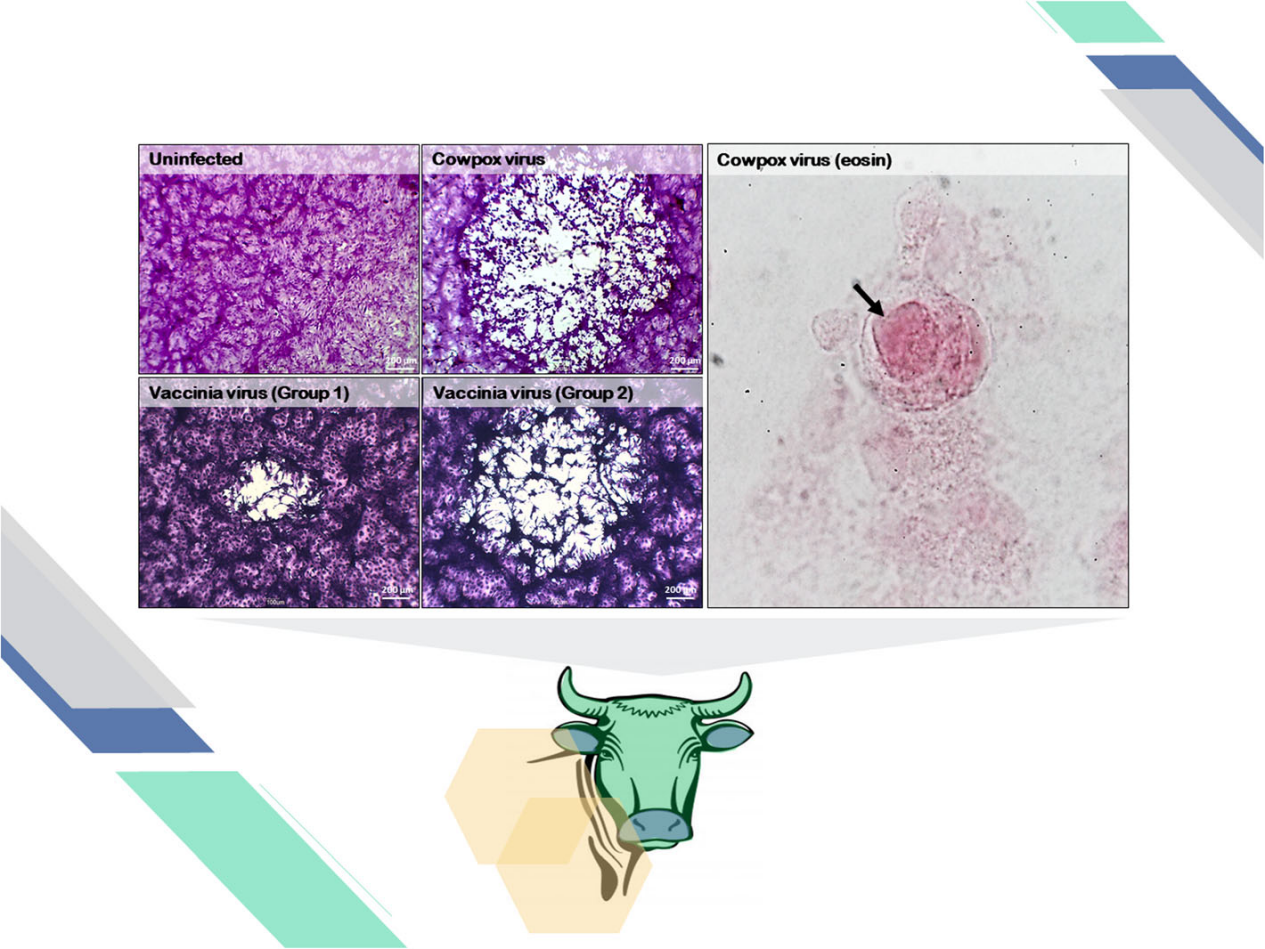
Cytopathic effects of orthopoxvirus in BSC-40 cells. Monolayers of cells infected or not infected with orthopoxvirus and stained with crystal violet. Visualization of inclusion corpuscle in an eosin-stained infected cell is also shown. Total magnification is 100x

**Fig. 3:**
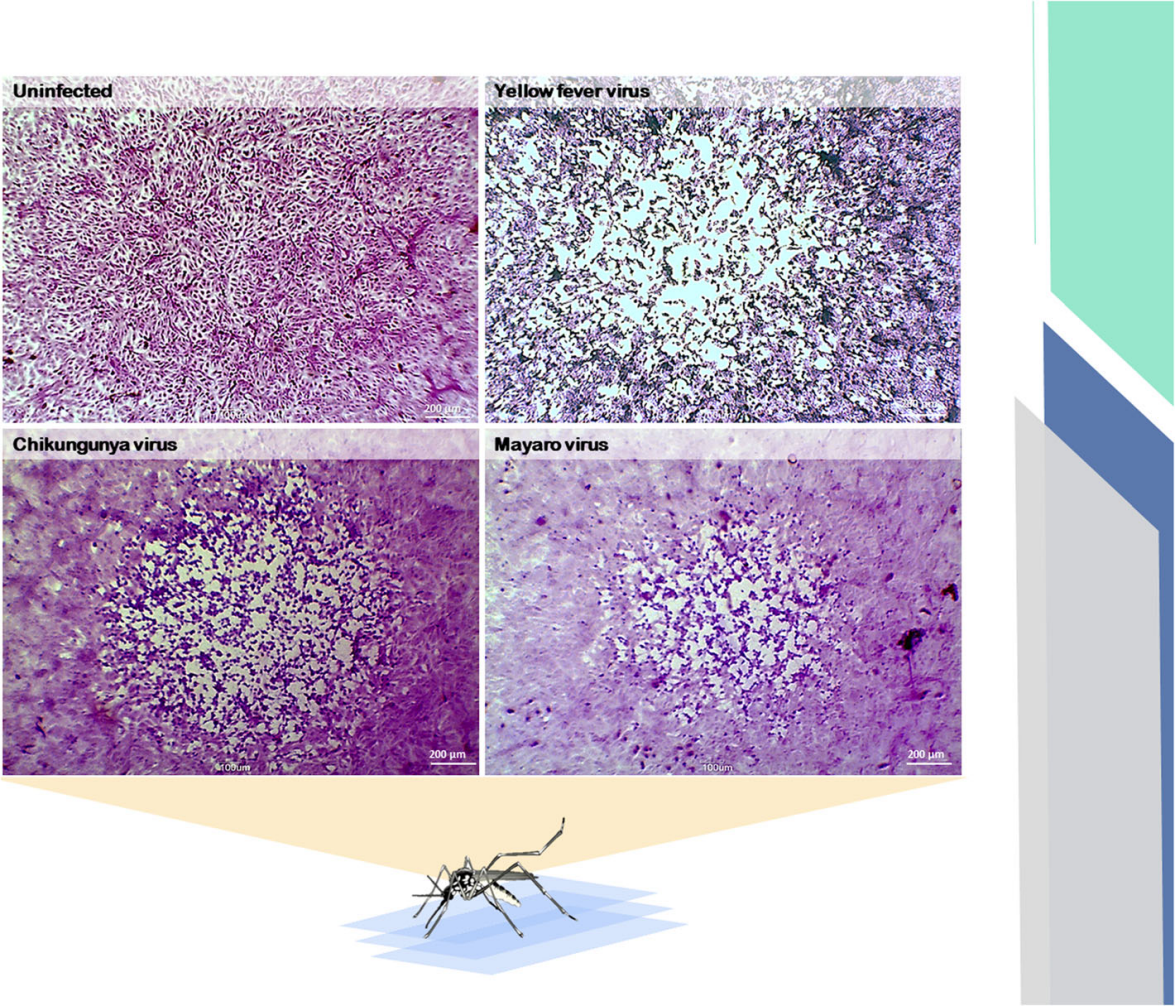
Cytopathic effects of arboviruses in Vero cells. Monolayers of cells infected or not infected with arboviruses (flavivirus and alphavirus) and stained with crystal violet. Total magnification is 100x

Two cell lines were infected with different viruses to produce CPE slides (Figs. 2 and 3). BSC-40 cells assays provided visibly distinct lysis plaques after infection with different viruses. CPXV generated large circular plaques of clear destruction with very stained round cell remains (Fig. 2). The presence of A-type eosinophilic inclusion bodies was also observed in CPXV-infected BSC-40 cells (Fig. 2). These inclusion bodies appeared as large pink circles in the host cell cytoplasm (Fig. 2, black arrow). Both VACV groups presented a distinct plaque phenotype; group I showed small lysis plaques, while group II showed large plaques with very stained and stretched cells that formed a web (Fig. 2). There were lysis plaques generated by arbovirus infection in the Vero cell monolayers. YFV generated largely undefined edge lysis plaques that included numerous stretched cells (Fig. 3). CHIKV presented large circular plaques, while MAYV showed small undefined edge lysis plaques (Fig. 3).

### “Virus Goes Viral” kit and associated teaching materials

**Fig. 4:**
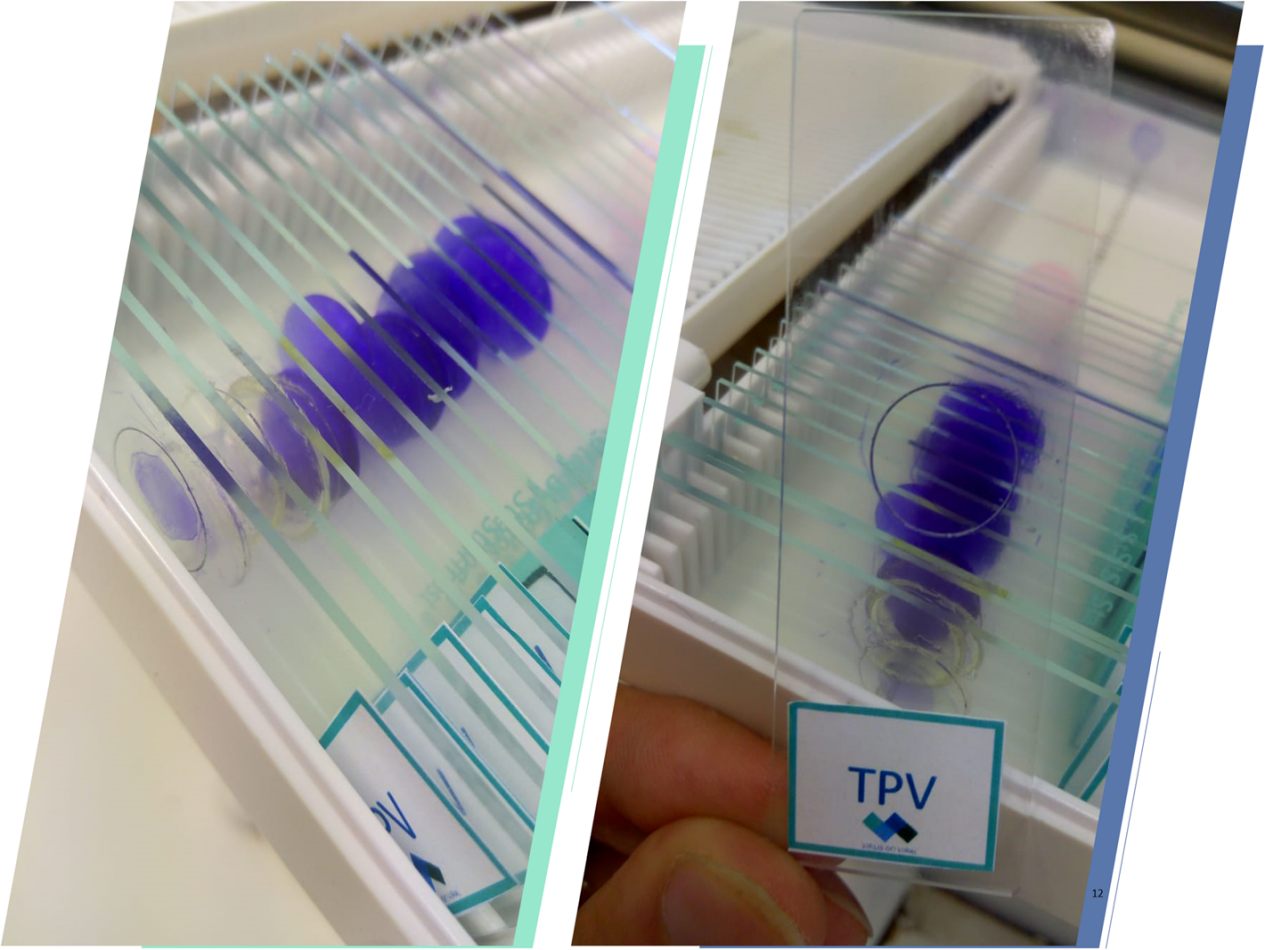
“Virus Goes Viral” kit. The stock of kit blades that highlights the individual labels

The slides generated in previous sections were used to compose the “Virus Goes Viral” kit. The kit has a total of 15 labeled slides: five viral particle slides, nine CPE slides, and one viral factory slide (Fig. 4). The kit includes associated teaching materials on a CD-ROM, including an infographic material, which aims to assist in understanding what is being observed (Additional file 3) and also high-resolution model images of each slide and CPE images from several giant viruses in *A. castellanii* and *V. vermiformis* that were not fixed and stained in slide form (Additional file 1: Figure S1 and Additional file 2: Figure S2). These CPEs include: cell rounding for CVG, Marseillevirus, PDV, TPV, and KAUV; cell stretching for OBRV; cell bunches for TPV; lysis plaques for FSTV (Additional file 1: Figure S1 and Additional file 2: Figure S2). The “Virus Goes Viral” kit and its attached material encompass 13 different viral species and 17 miscellaneous effects in host cells. This kit has been used in practical virology classes for the Biological Sciences course (Universidade Federal de Minas Gerais [UFMG], Brazil). The students seemed very receptive to the proposal and many were excited about the practice. Some expressed interest in starting research with giant viruses and initiated contact with our laboratory.

## Discussion

The field of virology has a long and strong history of educational innovations. The actual challenge is to develop specific initiatives that can be broadly implemented early in the curriculum ([26], see Table 1). Virology is a very important subject for several undergraduate courses. Students from medical and biological courses need to learn virology; however, costs and adequate facilities, including biological hoods in aseptic rooms, are frequently obstacles [3]. Although digital learning and other approaches to traditional texts or lectures increase student receptiveness, standard educational materials, such as microscope reference slides, are invaluable pedagogical resources [27, 28]. Two main goals led us to develop the “Virus Goes Viral” kit. The first goal was to foster better understanding among students about the basic concepts of virology. Achieving this goal requires only simple installations with one light microscope or computer, and these measures are congruent to the Brazilian reality.

The second goal was to introduce and spread knowledge about giant viruses to high school and undergraduate students. Our group has been dedicated to the exploration of giant viruses and isolates from different samples, including water, soil, sewage, and clinical samples, as well as in extreme environments, for example, permafrost and soda lakes [12, 17, 29, 30]. Giant viruses led biologists to rethink the nature of life. In recent years, giant viruses have been widely publicized in magazines, newspapers, blogs, television channels, and video sharing platforms; this coverage is an important example of scientific dissemination.

A common misconception in the general population is that all microorganisms are associated with human diseases [31]. Integrating giant viruses into the classroom represents a way to expand knowledge and dispel the notion that viruses are solely human pathogens. Indeed, giant viruses highlight that all organisms can be infected with viruses. Besides, giant viruses can represent tools to help the general public understand the importance of microorganisms, their evolutionary biology and ecology in the ecosystem, and their ancestry. With this material, we believe the educator can explore the revolutionary aspects of these viruses and create an inspiring learning environment. This is intended to be a simple but useful material for teaching virology. It can and should be associated with other educational strategies, including other hands-on lessons, according to the educator’s plan and institution resources.

Our kit covers 13 viral species, including vertebrate viruses, protozoan viruses, and arboviruses. This range of viral models illustrates for students the diversity of the virosphere. Besides, the CPEs covered by the kit are an important discussion point with students about specific interactions between virus and host. An interesting example of virus-host interactions is the mechanism of TPV and amoebas [32]. Virus-induced infected cells can aggregate with uninfected cells to form bunches that can increase TPV fitness [32]. These bunches are present in the “Virus Goes Viral” attached material. Another example is the VACV isolates that show a biological diversity that allows separating the viruses into different groups [33]. The VACV strain shows plaque phenotypes that differ in size; for example, the VACV group II has larger plaques than the VACV group I [33]. Our kit contains slides with VACV differentiation to make it clear to students that even within the same viral species there is variation.

## Conclusion

We designed a slide microscope kit named “Virus Goes Viral” that explores giant virus particles and some aspects of animal virus interactions with cells, with the aim to provide an innovative approach in virology teaching. Slides were produced by staining, with crystal violet, purified giant viruses and BSC-40 and Vero cells infected with viruses of the genera *Orthopoxvirus*, *Flavivirus*, and *Alphavirus*. The kit contains a slide for viral factory visualization in amoebas stained with the hemacolor kit as well as associated teaching materials on a CD-ROM, including high-resolution model images of each slide and CPE images of several giant viruses in *A. castellanii* and *V. vermiformis*. This kit has been used in practical virology classes for the Biological Sciences course (UFMG, Brazil), and it will soon be made available at a low cost to elementary school teachers in institutions that have microscopes. We hope this tool will foster an inspiring learning environment about virology.

## Supplementary information

**Additional file 1: Figure S1.** The cytopathic effects caused by different viruses on *Acanthamoeba castellanii.*

**Additional file 2: Figure S2.** The cytopathic effects caused by different viruses on *Vermamoeba vermiformis.*

**Additional file 3. **Infographics related to *Virus Goes Viral* educational kit.

## Data Availability

All relevant information is provided in this current manuscript. If required, the data presented in this work can be shared by e-mail.

## Abbreviations

APMV: *Acanthamoeba polyphaga* mimivirus
CHIKV: Chikungunya virus
CPEs: Cytopathic effects
CPXV: Cowpoxvirus
CVG: Cedratvirus
DNA: Deoxyribonucleic acid
FBS: Fetal bovine serum
FSTV: Faustovirus
MAYV: Mayaro virus
MOI: Multiplicity of infection
NCLDV: Nucleocytoplasmic large DNA viroses
NYMV: Niemeyer virus
OBRV: Orpheovirus brasiliensis
PAS: Phophate saline
PDV: Pandora virus
PYG: Peptone-yeast extract with glucose
TPV: Tupanvirus
VACV: Vaccinia virus
YFV: Yellow fever virus
