# Supplementary figures and images for: Virus goes viral: an educational kit for virology classes
Source: Virol J. 2020 Jan 31;17:13. doi: 10.1186/s12985-020-1291-9 (PMC6995199; doi:10.1186/s12985-020-1291-9)

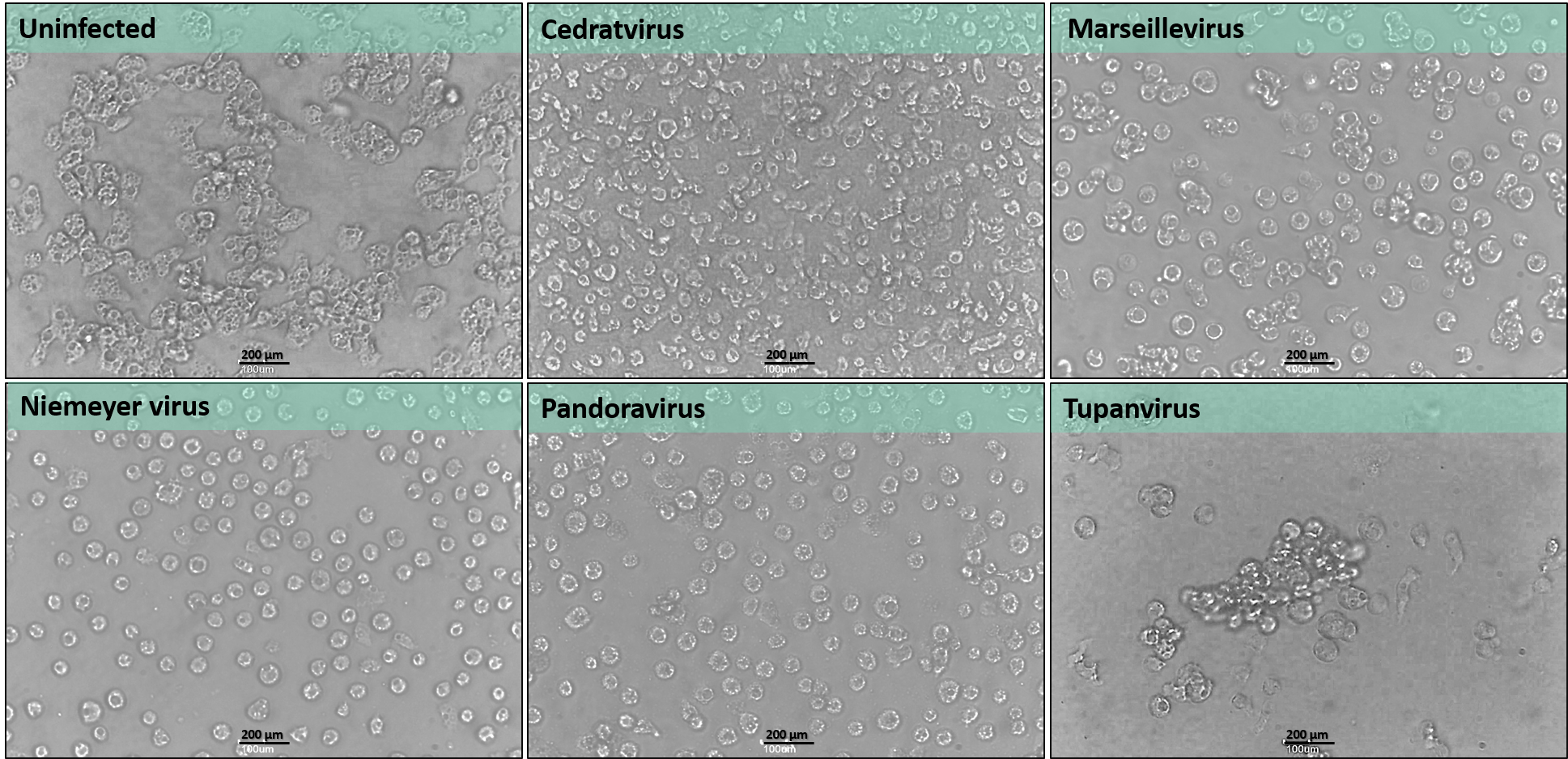

Supplement: Supplementary file 1 — Additional file 1: Figure S1. The cytopathic effects caused by different viruses on Acanthamoeba castellanii. [file 12985_2020_1291_MOESM1_ESM.tif]

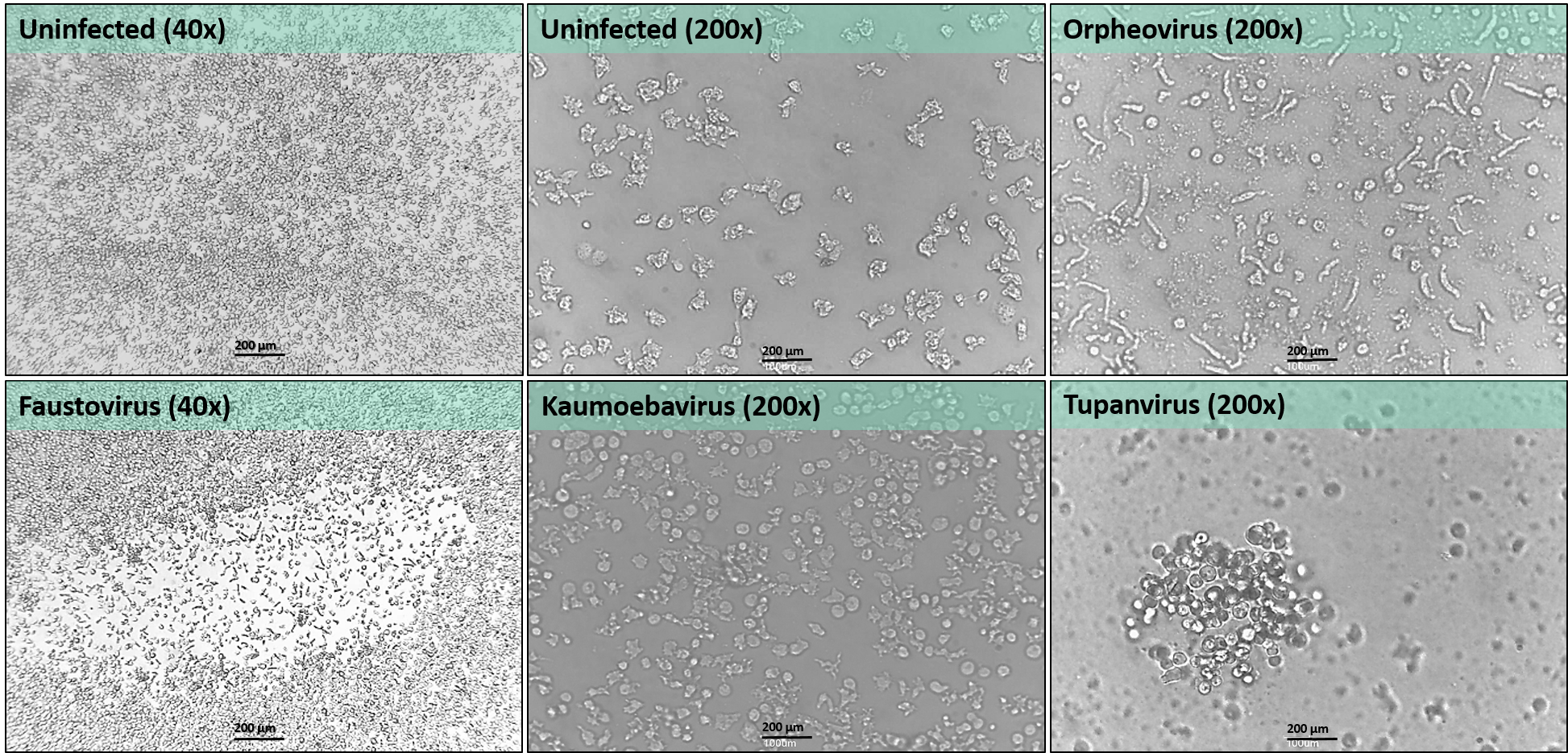

Supplement: Supplementary file 2 — Additional file 2: Figure S2. The cytopathic effects caused by different viruses on Vermamoeba vermiformis. [file 12985_2020_1291_MOESM2_ESM.tif]
