# Supplementary material for: Virus goes viral: an educational kit for virology classes
Source: Virol J. 2020 Jan 31;17:13. doi: 10.1186/s12985-020-1291-9 (PMC6995199; doi:10.1186/s12985-020-1291-9)

Virus Goes Viral Infographic

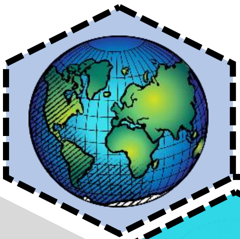

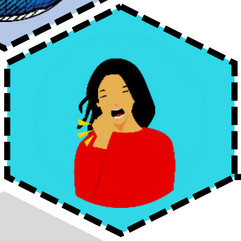

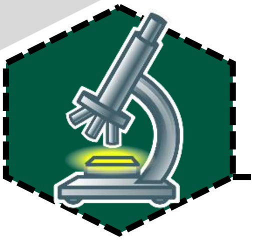

Abundance

Viruses are the most numerous entities on Earth, and they are found in the majority of ecosystems.

Pathogenic?

Although viruses are often associated with disease, not all are pathogenic. They represent strong forces of natural selection and population control for all living organisms.

Minor of minors?

Previously, viruses were considered to be the smallest group of organisms, however recent discoveries have shown the diversity of virus sizes. Some of them can even be seen from ordinary microscopes!

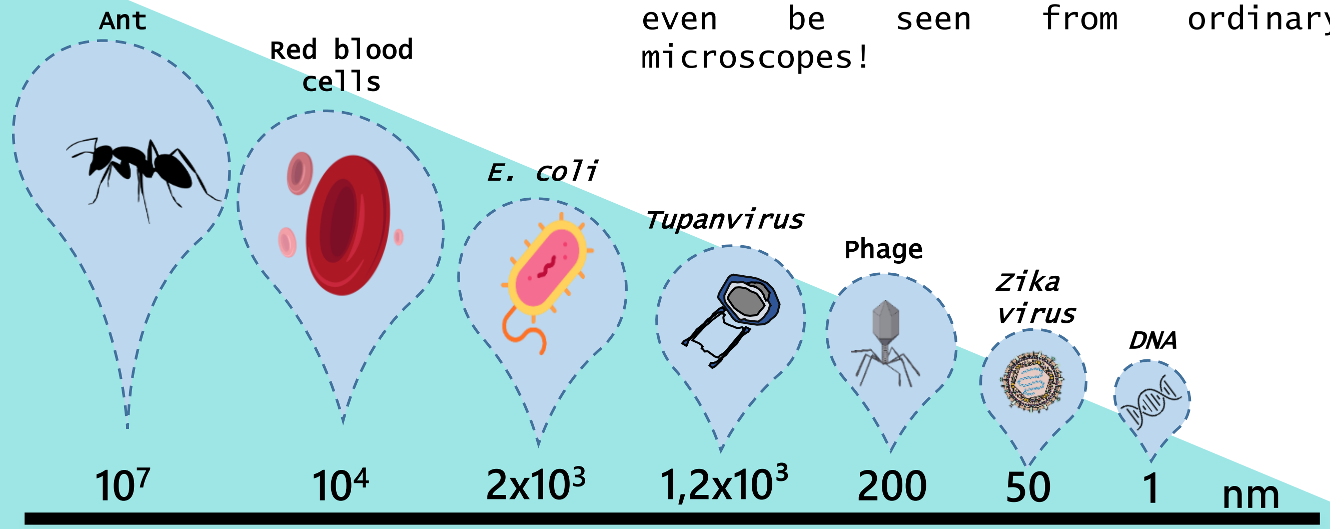

Virus Goes Viral Infographic

• Slides provided from the following viruses:

Vírus	Family	Genus	Genome Type	Genome size	Particle Size	Major Host	CPE
Cedratvirus	*Unassigned*	*Unassigned*	dsDNA	~590 kbp	1 µm	Free Living Amoebas	Cell rounding and nucleus loss
Chikungunya virus	*Togaviridae*	*Alphavirus*	ssRNA	11.8 kb	70 nm	Nonhuman primates and mosquitoes	Cytoplasmic blebbing, rounding, shrinkage and lysis
Cowpox virus	*Poxviridae*	*Orthopoxvirus*	dsDNA	~222 kbp	350-250 nm	Woodland rodents, cats, humans	Cell lysis (lysis plaque)
Mayaro virus	*Togaviridae*	*Alphavirus*	ssRNA	11.8 kb	70 nm	Nonhuman primates and mosquitoes	Cytoplasmic blebbing, rounding, shrinkage and lysis
Niemeyer virus	*Mimiviridae*	*Mimivirus*	dsDNA	~1.3 Mb	~750 nm	Free Living Amoebas	Cell rounding and nucleus loss
Orpheovirus	*Unassigned*	*Unassigned*	dsDNA	~1.4 Mb	1.3 µm	Free Living Amoebas	Tapering and increased motility
Pandoravirus	*Unassigned*	*Unassigned*	dsDNA	~2.5 Mb	1 µm	Free Living Amoebas	Cell rounding and nucleus loss
Tupanvirus	*Mimiviridae*	*Unassigned*	dsDNA	1.44 - 1.51 Mb	> 2 µm	Free Living Amoebas	Formation of bunches
Vaccinia virus	*Poxviridae*	*Orthopoxvirus*	dsDNA	~190 kbp	350-250 nm	Cattle, buffaloes, humans	Cell lysis (lysis plaque)
Yellow fever virus	*Flaviviridae*	*Flavivirus*	(+)sense RNA	~11 kb	40-50 nm	Non-human primates, humans, mosquitoes (*Aedes* and *Haemagogus* species)	Cell lysis (lysis plaque)

Diversity

Viruses are very diverse, come in many shapes, sizes, different genetic materials. Features selected throughout evolution to succeed in infecting their hosts.

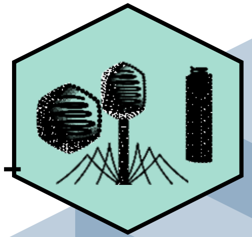

Virus Goes Viral Infographic

- **General cycle of replication**

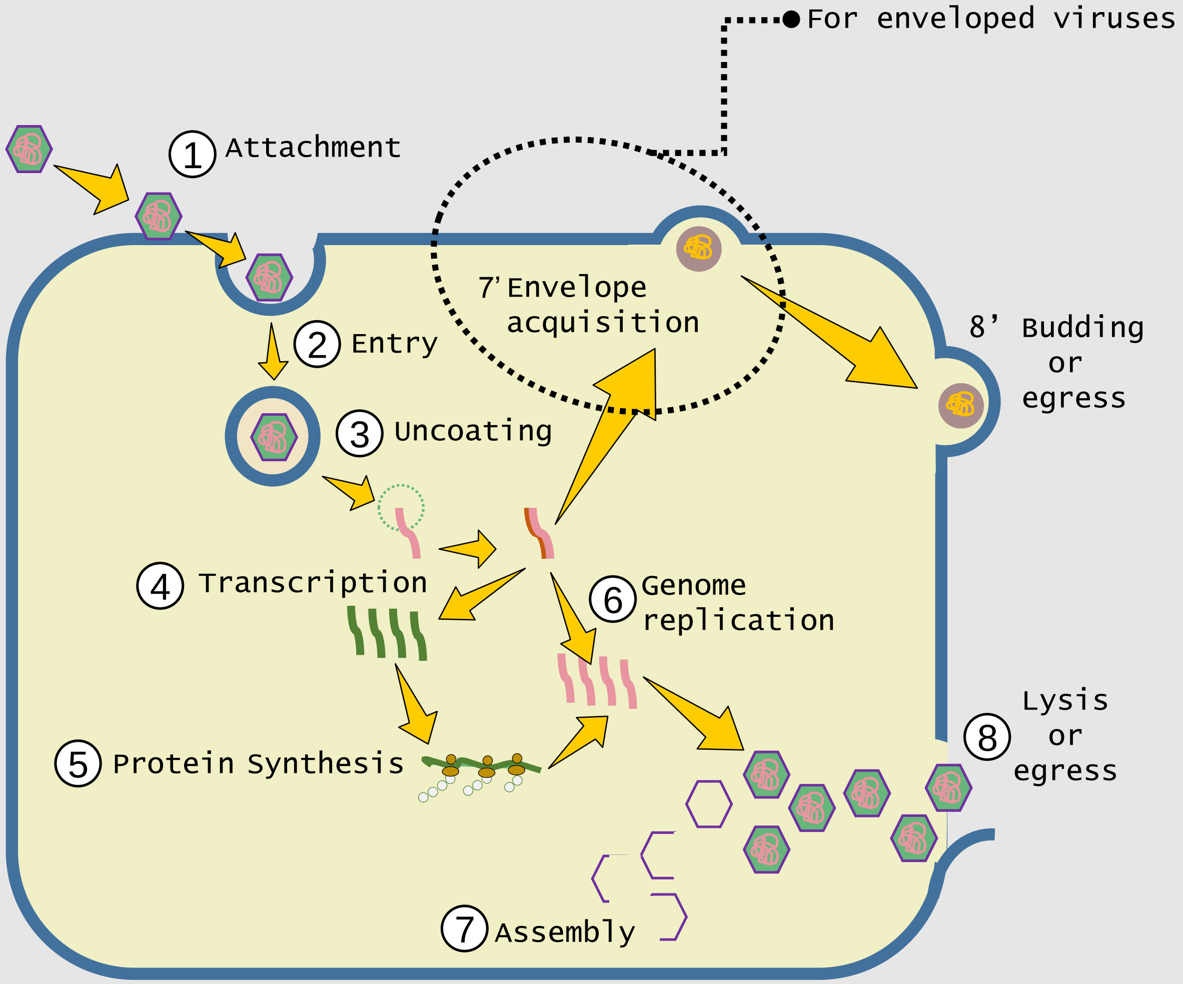

Intracellular parasites

Viruses are obligate intracellular parasites, so they use the machinery of infected cells to multiply. Although each virus may have its own peculiarities during its replication cycle, it usually goes through the steps described above.

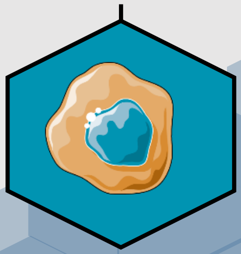

Virus Goes Viral Infographic

- **Virus-cell interaction: lysis plaques**

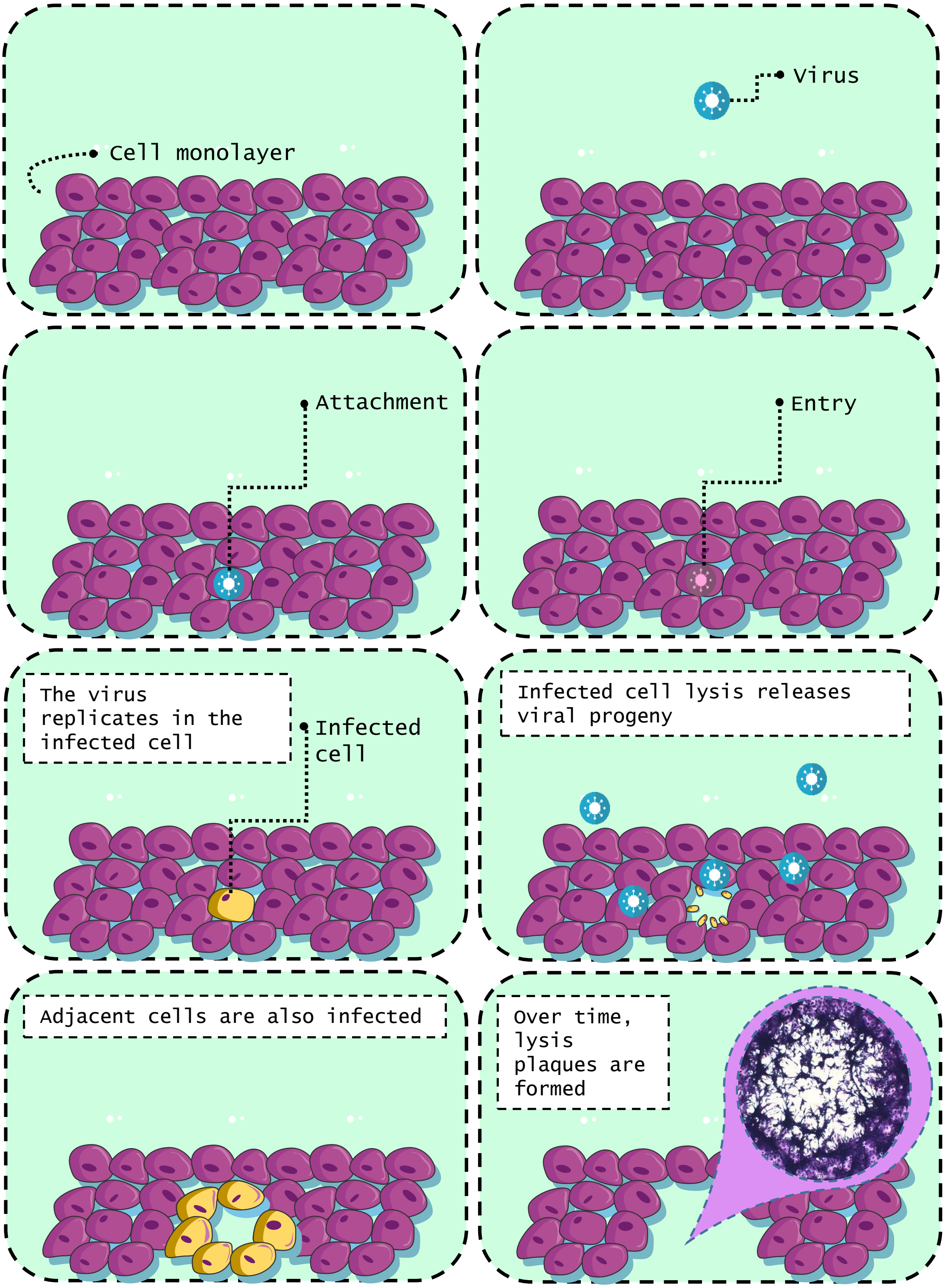

Supplement: Supplementary file 3 — Additional file 3. Infographics related to Virus Goes Viral educational kit. [file 12985_2020_1291_MOESM3_ESM.pdf]
